# The formal EU‐US Meniscus Rehabilitation 2024 Consensus: An ESSKA‐AOSSM‐AASPT initiative. Part I—Rehabilitation management after meniscus surgery (meniscectomy, repair and reconstruction)

**DOI:** 10.1002/ksa.12674

**Published:** 2025-05-12

**Authors:** Nicolas Pujol, Airelle O. Giordano, Stephanie E. Wong, Philippe Beaufils, Juan Carlos Monllau, Elanna K. Arhos, Roland Becker, Francesco Della Villa, J. Brett Goodloe, James J. Irrgang, Jitka Klugarova, Emma L. Klosterman, Aleksandra Królikowska, Aaron J. Krych, Robert F. LaPrade, Robert Manske, Nicky van Melick, Jill K. Monson, Marko Ostojic, Mark V. Paterno, Tomasz Piontek, Simone Perelli, Alexandre Rambaud, James Robinson, Laura C. Schmitt, Eric Hamrin Senorski, Thorkell Snaebjornsson, Adam J. Tagliero, C. Benjamin Ma, Robert Prill

**Affiliations:** ^1^ Department of Orthopedic and Trauma Surgery Centre Hospitalier de Versailles Le Chesnay‐Rocquencourt France; ^2^ Department of Physical Therapy University of Delaware Newark Delaware USA; ^3^ Department of Orthopaedic Surgery University of California San Francisco California USA; ^4^ ESSKA Office, Centre Médical, Fondation Norbert Metz Luxembourg Luxembourg; ^5^ Department of Orthopedics and Traumatology Hospital del Mar Barcelona Spain; ^6^ Department of Physical Therapy and Human Movement Sciences Northwestern University Chicago Illinois USA; ^7^ Department of Orthopedics and Traumatology, Brandenburg Medical School Theodor Fontane University Hospital Brandenburg/Havel Brandenburg Germany; ^8^ Faculty of Health Science Brandenburg, Brandenburg Medical School Theodor Fontane Potsdam Germany; ^9^ Faculty of Health Sciences Brandenburg, Brandenburg Medical School Theodor Fontane Brandenburg an der Havel Germany; ^10^ Education & Research Department Isokinetic Medical Group, FIFA Medical Centre of Excellence Bologna Italy; ^11^ Department of Orthopaedic Surgery University of Virginia Health System Charlottesville Virginia USA; ^12^ Department of Physical Therapy University of Pittsburgh School of Health and Rehabilitation Sciences Pittsburgh Pennsylvania USA; ^13^ Cochrane Czech Republic, Czech CEBHC: JBI Centre of Excellence, Czech GRADE Network Institute of Health Information and Statistics of the Czech Republic Prague Czech Republic; ^14^ Center of Evidence‐based Education and Arts Therapies: A JBI Affiliated Group, Faculty of Education Palacký University Olomouc Prague Czech Republic; ^15^ Michigan Medicine Department of Orthopaedic Surgery Ann Arbor Michigan USA; ^16^ Physiotherapy Research Laboratory University Centre of Physiotherapy and Rehabilitation, Faculty of Physiotherapy, Wroclaw Medical University Wroclaw Poland; ^17^ Mayo Clinic Department of Orthopedic Surgery Rochester Minnesota USA; ^18^ Twin Cities Orthopedics Edina Minnesota USA; ^19^ Wichita State University, Ascension Via Christi Wichita Kansas USA; ^20^ Sports & Orthopedics Research Center, Anna TopSupport Eindhoven the Netherlands; ^21^ Twin Cities Orthopedics Eagan Minnesota USA; ^22^ Osteon Orthopedics and Sports Medicine Clinic Mostar Bosnia and Herzegovina; ^23^ Division of Occupational Therapy and Physical Therapy, Division of Sports Medicine Cincinnati Children's Hospital Medical Center Cincinnati Ohio USA; ^24^ Department of Pediatrics University of Cincinnati College of Medicine Cincinnati Ohio USA; ^25^ Rehasport Clinic and Sport Medicine Lab, Department of Spine Disorders and Pediatric Orthopedics University of Medical Sciences Poznań Poland; ^26^ Knee and Arthroscopy Unit, Institut Català de Traumatologia i Medicina del'Esport (ICATME) – Hospital Universitari Dexeus Universitat Autònoma de Barcelona Barcelona Spain; ^27^ Institute of Physiotherapy of Saint‐Etienne – Saint‐Michel Campus Saint‐Etienne France; ^28^ MS Knee Specialists Bristol UK; ^29^ Division of Physical Therapy, School of Health and Rehabilitation Sciences and OSU Sports Medicine Research Institute Ohio State University Columbus Ohio USA; ^30^ Department of Health and Rehabilitation Institute of Neuroscience and Physiology, Sahlgrenska Academy, University of Gothenburg Gothenburg Sweden; ^31^ Landspitali, University Hospital of Iceland Reykjavik Iceland; ^32^ Medical Faculty University of Iceland Reykjavik Iceland

**Keywords:** consensus, knee, meniscus, physical therapy, rehabilitation, repair

## Abstract

**Purpose:**

The aim of part one of this EU‐US consensus was to combine literature research and expertise to provide recommendations for the usage of rehabilitation (including physical therapy) of patients undergoing surgical treatment for degenerative meniscus lesions or acute meniscus tears (including meniscectomy, repair, or reconstruction). Prevention programmes, non‐operative treatment of acute tears and degenerative lesions, return to sports and patient‐reported outcome measures will be presented in a part II article.

**Methods:**

This consensus followed the *European Society for Sports Traumatology and Arthroscopy* (ESSKA)'s ‘formal consensus’ methodology. For this combined ESSKA, *American Orthopedic Society for Sports Medicine* and *American Academy of Sports Physical Therapy* initiative, 67 experts (26 in the steering group and 41 in the rating group) from 14 countries (US and 13 European countries), including orthopaedic surgeons, sports medicine doctors and physiotherapists were involved. Steering group members established guiding questions, searched the literature and proposed statements. Rating group members assessed the statements according to a Likert scale and provided grades of recommendations, reaching a final agreement about rehabilitation of the knee after meniscus surgery. Final documents were then assessed by a peer review group to address the geographical adaptability.

**Results:**

The overall level of evidence in the literature was low. Of the 19 questions (leading to 29 statements), 1 received a Grade A of recommendation, 2 a Grade B, 9 a Grade C and 17 a Grade D. Nevertheless, the mean median rating of all questions was 8.2/9 (9 being the highest rating on a scale of 1–9). The global mean rating was 8.4 ± 0.2, indicating a high agreement. Rehabilitation depends on the type of lesion, the treatment performed and is the same after medial or lateral meniscus surgery. Rehabilitation after meniscectomy should follow a criterion‐based rehabilitation protocol, based on milestones rather than a time‐based protocol. After meniscus repair and reconstruction, rehabilitation should be progressed according to both time and criterion‐based milestones.

**Conclusion:**

Rehabilitation after meniscus surgery is a debated topic that may influence surgical outcomes if not optimally performed. This international formal consensus established clear, updated and structured recommendations for both surgeons and physiotherapists treating patients after meniscus surgery.

**Level of Evidence:**

Level I, consensus.

**Abbreviations**
AASPT: American Academy of Sports Physical Therapy
ACL: anterior cruciate ligament
ACLR: anterior cruciate ligament reconstruction
AOSSM: American Orthopaedic Society For Sports Medicine
CKC: closed kinetic chain
ESSKA: Eurpoean Society of Sports Traumatology, Knee Surgery and Arthroscopy
FWB: full weight‐bearing
LOE: level of evidence
MAT: meniscal allograft transplantation
NEMS: neuromuscular electrical stimulation
PWB: partial weight‐bearing
ROM: range of motion
RTP/RTS: return to sports
WB: weight‐bearing

## INTRODUCTION

Meniscus surgery is one of the most common procedures performed worldwide. The indications of treatment and practice have evolved with time due to the improved knowledge of the biomechanics of the meniscus and the consequences of extensive meniscectomies [11]. Recent studies focus on the indications for surgery, and if needed, which surgery preserves as much meniscus tissue as possible while providing the best short‐ and long‐term functional results.

Following these trends, the *European Society for Sports Traumatology and Arthroscopy* (ESSKA) initiated the European Meniscus Consensus Project in 2016 [2]. The aim of the first part was to provide a reference document for the management of degenerative meniscus lesions (DMLs), based both on scientific literature and balanced clinical expertise. Following this, arthroscopic partial meniscectomy became not a first but a second‐line treatment for treating DML. In 2019, the second ESSKA meniscus consensus [13] provided recommendations for the surgical treatment of Traumatic meniscus tears. The main message of this second consensus was that preservation of the meniscus (including repair or partial meniscectomy) should be the first treatment philosophy for traumatic tears. Post‐operative rehabilitation after meniscus surgery may vary with time, location/country, expertise and habit. Detailed rehabilitation decisions were not covered by the previous consensuses.

For anterior cruciate ligament (ACL) reconstruction, robust rehabilitation protocols and recommendations exist [6, 9]. For the meniscus, literature is still scarce [5, 12, 19]. While many indications and techniques of meniscus surgery are made based on sufficient evidence, many surgeons, sports medicine doctors and physiotherapists still face multiple options where recommendations are lacking, especially for the post‐operative care after meniscus surgery. That is the reason why ESSKA, the American Orthopedic Society for Sports Medicine (AOSSM) and the *American Academy of Sports Physical Therapy* (AASPT), three main scientific societies working around meniscus pathology and rehabilitation, decided to cover the topic together in order to give unified messages.

The aim of this formal consensus was to provide recommendations for the usage of rehabilitation (including physical therapy) for patients with symptomatic menisci. The first part of the consensus will focus on rehabilitation performed after any surgical treatment for DMLs, traumatic meniscus tears (meniscectomy or repair) or meniscus reconstruction.

The second part of the consensus will cover prevention, non‐operative treatment and return to sports (RTS) modalities. The full version of the consensus project can also be read in more detail on the ESSKA website (https://esskaeducation.org/esska-consensus-projects) and on the ESSKA Academy Website (Open Access) https://esskaeducation.org/sites/default/files/2024-07/The%20formal%20EU-US%20Meniscus%20Rehabilitation%20Consensus.pdf.

## MATERIALS AND METHODS

An EU‐US consensus project was established by ESSKA‐AOSSM and AASPT between 2022 and 2024, focusing on the rehabilitation management of meniscus tears. Prevention, non‐operative and post‐operative management for both DMLs or acute tears (meniscectomy, repair and reconstruction), RTS and patient‐related outcomes were considered in this project.

The process and methodology of this consensus project were similar to previously published ESSKA consensus projects [3, 4]. Chairs were nominated by the ESSKA board. A steering group was built with experts in the field of meniscus and rehabilitation. The steering group was divided into two groups, one being mainly responsible for developing questions and statement drafts (question group) and the other one for screening the current literature and providing evidence to support the development of statements (literature group).

The consensus equally involved orthopaedic surgeons, physiotherapists and sports medicine practitioners, who were equally distributed among the United States and Europe and were equally selected by AOSSM and AASPT. First, the question group queried relevant topics in the field. According to the questions, literature was searched and summarized by the literature group. This comprehensive search was performed on MEDLINE (Pubmed), Web of Science and SCOPUS databases without time constraints for the search. All clinical studies were included (randomized and non‐randomized clinical trials, multicenter studies, reviews, systematic reviews and meta‐analyses). The process was restricted to the English language. Search String was validated by the group and is provided as Supplement 1.

Overall, 395 relevant papers were screened and included, and additional literature was manually searched. Each bibliography of the full‐text articles was screened by hand for inclusion. Any disagreements were discussed and settled between the members of the literature group. After reviewing the literature, the question/statement group formulated recommendation statements to answer the agreed‐upon questions and added grading according to the level of evidence (LOE) supporting the statements. Grade A was defined as a high level of scientific support, Grade B as a scientific presumption, Grade C as a low level of scientific support, and Grade D as an expert opinion. After recommendations were completed, an independent rating group rated the statements according to scientific quality and clinical experience using the Likert scale (ranging from 1 to 9 [i.e., totally inappropriate to totally appropriate]). The final agreement was classified as high (median value ≥7, and the ratings were all ≥7) or Relative (median value ≥7, all scores >5).

At the end of the first round, 19 questions (29 statements overall) focusing on rehabilitation after meniscus surgery were formulated by the steering group. A revised manuscript draft was prepared and resubmitted to the rating group for a second assessment for any statements where the median agreement was below 7. The same level of agreement was applied for the second round. A combined steering‐rating group meeting allowed us to finalize the last version of the document.

**Figure 1:**
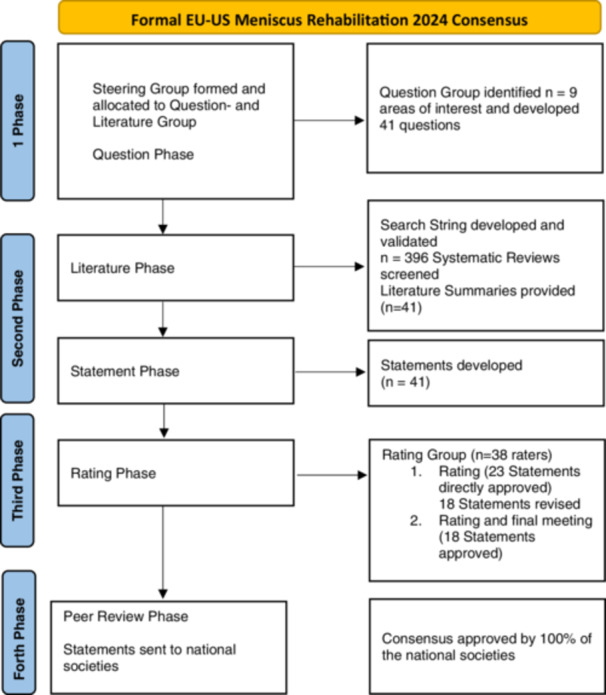
Flowchart.

The last version of the manuscript was finally reviewed by representatives of the affiliated scientific societies of ESSKA, AOSSM and AASPT to produce the final consensus statement. The aim of this last step was to check the geographic adaptability and clinical clarity for worldwide use. The process is displayed in a flowchart (Figure 1).

## RESULTS

Of the 19 questions (leading to 29 statements), 1 received a Grade A rating, 2 a Grade B, 9 a Grade C and 17 a Grade D. This means that low to moderate scientific LOE was available in the current literature for making most of the statements and the weight of clinical expertise in the statements set up. Six questions went on to a second revision and rating. Overall, the mean median rating was 8.2/9 (8–9), and the global mean rating was 8.3 ± 0.2/9, meaning that the global agreement between experts was high.

### Rehabilitation management after partial meniscectomy

1. What rehabilitation treatment is best indicated for the management of patients after isolated partial meniscectomy? Is there an evidence‐based treatment protocol?

No evidence‐based treatment protocol exists. A criterion‐based rehabilitation protocol, based on milestones, is recommended. Following partial meniscectomy, immediate full weight‐bearing (FWB) and full range of motion (ROM) are permitted as tolerated per symptoms (Grade C).

Although large effusions are uncommon after partial meniscectomy, they occasionally do occur and can lead to significant quadriceps inhibition that may require the use of an assistive device, particularly in older people and those with high body mass index or other comorbidities (Grade D).

To address quadriceps strength and neuromuscular control deficits, the use of neuromuscular electrical stimulation (NMES), open kinetic chain and closed kinetic chain (CKC) strengthening is recommended as is seen in similar patient populations (i.e., after ACL reconstruction) (Grade C). Mean 8.4 ± 1.45, Median 8 (5–9), Relative agreement.

2. Is there a difference between rehabilitation proposed after medial or lateral partial meniscectomy?

There are no specific rehabilitation protocols after medial or lateral partial meniscectomy. More adverse events (persisting swelling and pain, risk of early chondrolysis) may occur after lateral partial meniscectomy, which may result in delayed return to higher‐impact activities and sports compared to medial partial meniscectomy (Grade D).

Agreement: Mean 7.8 ± 1.36, Median 8 (5–9), Relative agreement.

3. Is there any difference between traumatic meniscus tears and DMLs in terms of rehabilitation after partial meniscectomy?

There is no comparative data to support any difference between rehabilitation protocols for partial meniscectomy for degenerative lesions and traumatic tears. Rehabilitation protocols can vary based on patient factors and the status of the knee post‐operatively. Partial meniscectomy for degenerative lesions may require slower rehabilitation progression (Grade D).

Agreement: Mean 8.3 ± 1.51, Median 8 (5–9), Relative agreement.

4. Should non‐WB or partial weight‐bearing (PWB) be recommended after arthroscopic partial meniscectomy?

After arthroscopic partial meniscectomy FWB is allowed early after surgery (Grade A).

Crutches may be recommended for mobility until gait is normalized (Grade D). Agreement: Mean 8.4 ± 1.00, Median 8 (5–9), Relative agreement.

5. For how long is rehabilitation recommended after arthroscopic partial meniscectomy? What persisting signs and/or symptoms during rehabilitation require a referral to a surgeon (for example, includes time from injury or surgery, ROM, pain, swelling, and activity limitations)?

There are multiple rehabilitation guidelines for return to walking, work and sport, with timeframes often ranging between 4 and 12 weeks. However, rehabilitation after arthroscopic partial meniscectomy should be criterion‐based and not time‐based. Return to these activities requires meeting progressive milestones throughout rehabilitation (e.g., effusion, ROM, quadriceps strength and neuromuscular control) and not just meeting healing time frames (Grade B).

Patients should be referred to an orthopaedic surgeon in cases of persistent pain, recurrence of stiffness and/or effusion, persistent functional instability, mechanical symptoms, any neurological symptoms, suspicion of infection or distal veinous thrombosis (DVT) (Grade B).

The inability to reach clinical milestones related to knee symptoms indicates a referral to the orthopaedic surgeon (Grade D).

Agreement: Mean 7.8 ± 1.02, Median 8 (5–9), Relative agreement.

### Rehabilitation management after meniscus repair

1. What rehabilitation treatment is best indicated for the management of meniscal repair? Is there an evidence‐based treatment protocol?

Whilst no evidence‐based rehabilitation protocol has been shown to be superior to another, post‐operative rehabilitation should consider the meniscal tear pattern and zone of injury, tissue quality and vascularity, surgical repair technique, repair stability, and any patient‐specific characteristics known to influence the surgical prognosis (Grade D).

For isolated meniscal repair, no evidence exists for the use of specific rehabilitation protocol or adjuvant therapies. For knee surgeries where there is concomitant meniscal repair NMES, in the early post‐operative phase, may help in the recovery of quadriceps activation (Grade D).

Combined time and criterion‐based protocols can be recommended. Management of effusion should be addressed in the protocol (Grade D).

Rehabilitation should be progressed according to both time‐ and criterion‐based milestones. A minimum of 4 months of rehabilitation may be recommended for repaired vertical tears of the meniscus, whereas complex, complete radial/root and horizontal tears may require a longer duration of rehabilitation (i.e., 6–9 months) (Grade D).

Agreement: Mean 8.3 ± 1.59, Median 8 (5–9), Relative agreement.

2. How does the location of meniscus tear/repair (i.e., stable, unstable, root tear, complex tear and white zone tear) influence the progression of rehabilitation after repair (i.e., ROM, WB and loading activities)?

For vertical longitudinal tears, FWB may be recommended, with a limitation of ROM for 6 weeks. For ramp lesions, the rehabilitation protocol is driven by associated procedures (e.g., ACL reconstruction) (Grade C).

Limitation of WB and ROM for 4–6 weeks is recommended for complex, horizontal, radial and root repairs (Grade C).

Following repair‐specific early post‐operative restrictions in ROM and WB, rehabilitation after meniscus repair should follow criterion‐ and time‐based components. Return to activities requires meeting progressive milestones throughout rehabilitation (e.g., effusion, ROM, quadriceps strength, neuromuscular control) and meeting healing time frames. This is different from arthroscopic partial meniscectomy, which is criteria‐based (Grade D).

Agreement: Mean 7.2 ± 1.96, Median 8 (5–9), Relative agreement.

3. Are there specific exercises that should be avoided following all meniscus repair (e.g., deep loaded flexion—deep squats)? If so, then for how long?

Deep squatting, jumping (deep loaded flexion) and rotational knee movements activities should be avoided for a minimum of 4 months. For vertical longitudinal tears, mini squats up to 30° can be recommended from Weeks 4 to 8, up to 45° from Weeks 8 to 12 and up to 60–90° from Weeks 13 to 16 (Grade D).

Agreement: Mean 7.6 ± 1.34, Median 8 (5–9), Relative agreement.

4. Does a medial and lateral repaired meniscus tear require different protocols?

Medial and lateral meniscal repairs may be rehabilitated similarly, with the tear type (radial, root and vertical) influencing rehabilitation rather than laterality (Grade C).

Agreement: Mean 7.8 ± 1.70, Median 8 (5–9), Relative agreement.

5. For how long rehabilitation is recommended after meniscus repair?

Rehabilitation after meniscus repair should be both criterion‐ and time‐based according to the healing process. A minimum of 4 months of rehabilitation may be advised for repaired vertical meniscus tears. Complex, radial, root and horizontal tears may require longer rehabilitation, up to 6–9 months (Grade D).

Agreement: Mean 8.5 ± 1.20, Median 8 (5‐9), Relative agreement.

6. What are the criteria‐based recommendations after meniscus repair?

Rehabilitation following meniscal repair should be divided into protective, restorative, and preparation to return to activity and sports phases, with additional criterion‐based goals recommended. Criteria for progression to the restorative phase of rehabilitation include full or nearly full passive ROM, no effusion, and neuromuscular control of the quadriceps. Initiation to return to the activity phase of rehabilitation begins once the patient demonstrates full active ROM, strength (larger or equal to 80% of the contralateral leg would be ideal), and adequate single‐leg dynamic knee control. Progression of quadriceps strength is recommended to be tested at each phase of rehabilitation via the use of isokinetic or appropriately stabilized handheld dynamometry (Grade D).

Agreement: Mean 7.9 ± 1.44, Median 8 (5–9), Relative agreement.

7. Does concomitant ACL reconstruction have an impact on rehabilitation after medial or lateral meniscus repair when compared to meniscus repair on a stable knee?

Rehabilitation protocols for repaired menisci with concomitant ACL reconstruction are similar to protocols for isolated meniscal repairs; however, RTS may be delayed on account of the ACL reconstruction. Meniscus repairs requiring limitation of WB and/or ROM would affect ACL rehabilitation protocols. Most stable vertical meniscal tear repairs do not affect ACL rehabilitation protocols (Grade C).

Agreement: Mean 8.1 ± 1.39, Median 9 (6–9), Relative agreement.

8. Questions for all repaired meniscus lesions

– Should non‐WB or PWB be recommended after meniscus repair (for lateral or medial meniscus)? If yes, for how long?
– If FWB is allowed, is the usage of crutches recommended after meniscus repair? If so, for how long crutches should be used?
– Is there any restriction in terms of ROM in the post‐operative period?
– Is there any indication for a knee brace after meniscus repair in the post‐operative period (locked brace or soft brace)?
– There are different categories of repaired traumatic meniscus tears, and a specific rehabilitation protocol should be considered for each of them (Grade C).

Agreement: Mean 7.3 ± 1.42, Median 8 (5–9), Relative agreement.

Two summary tables are provided (Tables 1 and 2).

**Table 1:** Synthesis of rehabilitation after meniscus repair, meniscectomy and meniscus reconstruction (Part 1).

		Weight‐bearing (WB)	Crutches	Range of motion (ROM) restriction	Knee brace
After meniscus repair	Stable vertical meniscal tear	Full WB	No	No	[a]
	Complex vertical meniscal tear repairs	Full WB	Yes	Yes	[a]
	Complete oblique and radial tears	No WB for 4 to 6 weeks	Depending on WB	0–90° for 4–6 weeks	[a]
	Horizontal lesions in the young athlete	Partial or no WB for 4 weeks	Depending on WB	0–90° for 4 weeks	[a]
	Ramp lesions	[a]	[a]	[a]	[a]
	Root tears	No WB for 6 weeks	Depending on WB	0–90° for 4 weeks	[a]
After meniscectomy		Full WB	Until gait is normalized	No	No
After meniscus reconstruction		No WB for 6 weeks	Yes	0–90° for 6 weeks	[a]

a No Recommendation: The Consensus group has no specific recommendation to advise.

**Table 2:** Synthesis of rehabilitation after meniscus repair, meniscectomy and meniscus reconstruction (Part 2).

	Phases	Criterion‐based rehabilitation	Duration of rehabilitation	
After meniscus repair	Protective phase	Until nearly full passive ROM, no effusion and neuromuscular control of the quadriceps	4 months (at least)	Ramp lesions
				Stable vertical meniscal tear
	Restorative phase	until Full ROM, >80% muscle strength, adequate single‐leg dynamic knee control	6–9 months	Complex vertical meniscal tear repairs
	Return to activity and sports phase	Time‐ and criterion‐based rehabilitation protocols (see chapter return to sports [RTS])		Complete oblique and radial tears
				Horizontal lesions in the young athlete
				Root tears
After meniscectomy	Return to these activities requires meeting progressive milestones throughout rehabilitation (e.g., effusion, ROM, quadriceps strength, neuromuscular control) and not just meeting healing time frames.	Referral to surgeon if: persistent pain, recurrence of stiffness and/or effusion, persistent functional instability, mechanical symptoms, any neurological symptoms, suspicion of infection or DVT, inability to reach clinical milestones related to knee symptoms	4–12 weeks	
After meniscus reconstruction	Depending on associated procedures	Time‐ and criterion‐based rehabilitation protocols	At least 9 months	RTS at least 12 months

Abbreviation: DVT, distal venous thrombosis.

### Meniscus reconstruction (transplantation or scaffold)

1. Is there any difference between different types of meniscus reconstruction (transplantation or scaffold) in terms of rehabilitation management?

Scaffolds and Allografts rehabilitation protocols follow the same rules and restrictions (Grade D).

Agreement: Mean 7.6 ± 1.82, Median 8 (5–9), Relative agreement.

2. What rehabilitation protocol is best indicated for the management of meniscus reconstruction (transplantation or scaffold)?

Rehabilitation after meniscus reconstruction should be criterion‐ and time‐based. Different stages of rehabilitation are an early protective phase, followed by an intermediate restorative phase, and finally a return to activity phase. Progression through phases will vary significantly for each patient based on associated procedures, level of activity and individual rates of recovery. Rehabilitation protocols could follow those of meniscus root repair (two roots are repaired after MAT), which require up to 9 months (or more) of rehabilitation. The consensus group suggests RTS at 12 months or more (Grade D).

Agreement: Mean 8.4, Median 9 (7–9), Strong agreement.

3. Is there a difference between rehabilitation proposed after medial or lateral meniscus reconstruction (transplantation or scaffold)?

Similar rehabilitation protocols can be used for medial or lateral meniscus reconstruction (Grade D).

Agreement: Mean 7.9 ± 1.00, Median 8 (6–9), Relative agreement.

4. What are the WB precautions recommended after meniscus reconstruction (transplantation or scaffold)?

Early WB has been shown to increase the risk of meniscus extrusion after meniscus reconstruction. Therefore, non‐WB is recommended for 6 weeks, progressing to FWB gait after 8 weeks. WB status may also be affected by other concomitant procedures such as osteotomy, cartilage or ligament surgeries. Weight‐bearing through a flexed knee (e.g., stairs, squatting and lunging) should be delayed. Initial WB should occur in relative knee extension, such as with gait and standing (Grade C).

Agreement: Mean 8.1 ± 1.46, Median 9 (7–9), Strong agreement.

5. Should the ROM be restricted in the post‐operative period after meniscus reconstruction (transplantation or scaffold)? If yes, for how long?

90° of non‐WB flexion should not be exceeded until 6 weeks post‐operatively. Associated surgical procedures may alter further ROM restrictions (Grade D).

Agreement: Mean 8.1 ± 1.21, Median 8 (6–9), Relative agreement.

6. Is a brace useful after meniscus reconstruction (transplantation or scaffold) in the post‐operative period (postop/unloading or functional for return to activity)?

There is a lack of evidence for bracing after meniscus reconstruction. The consensus group has no recommendation on the use of a knee brace. The use of a knee brace after meniscus reconstruction surgery is dependent on surgeon preference and concomitant procedures (Grade D).

Agreement: Mean 8.3 ± 1.11, Median 9 (6–9), Relative agreement.

## DISCUSSION

The most important message of this consensus is that moderate to low scientific LOE was available in reference to rehabilitation after meniscus surgery. Meniscus pathology is common; however, it is not often studied in isolation. Nevertheless, our recommendations are based on this literature and on the input of experts of three different specialities (orthopaedic surgeons, physiotherapists and sports physicians) from two continents (Europe and the United States), with a good agreement of the proposed statements.

The primary outcome of the consensus indicated that any patients undergoing meniscus surgery would benefit from rehabilitation. Rehabilitation protocols vary according to the type of surgery performed, and more research is needed specific to meniscus rehabilitation.

### After meniscectomy

Knee pain, lack of mobility, effusion, and quadriceps weakness are the most common clinical findings after a partial arthroscopic meniscectomy procedure. Regaining quadriceps strength is of utmost importance [22].

So, a criterion‐based rehabilitation protocol, based on milestones, is recommended. A systematic review that included 18 randomized controlled trials, out of which 6 were pooled in meta‐analysis, was published in 2013 [7] and highlighted already this point.

When comparing outpatient physical therapy with home exercise to home exercise alone, significant improvement was found in favour of the outpatient physical therapy group for the outcome of patient‐reported knee function on the Lysholm questionnaire, and for the knee flexion ROM. Expert opinion advocated that the treatment should include outpatient care and a home exercise programme. The following interventions are advised: early WB, progressive knee mobilization exercises, quadriceps and hamstring strengthening exercises (isometric and dynamic), sensory‐motor training, thermotherapy and an early return to activities. It may use adjuvants such as NMES, electromyography biofeedback and isokinetics [7].

### After meniscus repair

Rehabilitation should be adapted to the type of repair lesion, whatever the meniscus treated (lateral or medial) and whatever the status of the ACL (reconstructed or stable), following time‐based (to favour meniscus healing) and criterion‐based protocols.

Regarding root tear repair, there have been no long‐term studies comparing the efficacy of varying rehabilitation protocols for patients recovering from meniscal root repairs, especially regarding the time course for introducing WB, increasing ROM and beginning strength training. Nonetheless, biomechanical models of cyclic loading have underscored the need for more gradual and cautious rehabilitation protocols compared with other types of meniscal lesions [16, 21].

With radial/root repairs of meniscus, a strict emphasis on non‐WB should be implemented for the first 6 weeks, during which time, ROM should also be limited to 90 degrees [17].

One systematic review evaluated the management of ramp lesions. Seven studies have been analyzed, two RCTs and five retrospective case series. At least 2 weeks of non‐WB were indicated in all the studies, and FWB was permitted between 4 and 12 weeks. All authors allowed immediate post‐operative passive joint movements from 0° to 90° to avoid stiffness. At 6 weeks after surgery, a full ROM was permitted. The use of brace was prescribed in 181 (41%) of cases [1]. The literature with higher levels of evidence seems to support WB as tolerated, with no specific indications about crutch weaning [15, 18, 23].

Even individually analyzing all the papers of a systematic review about the comparison of accelerated and conservative rehabilitation protocols, we cannot draw strong conclusions. Some authors do not specify details on crutch use, and when it is specified, there are no clear indications about the duration of use [23]. Nevertheless, the recommendations of the consensus group are summarized in Table 1.

Concerning longitudinal/vertical tears, the literature [15, 18, 23] supports free motion as tolerated as soon as possible after surgery. Deep squats may be restricted initially. No standardized indications were found about the use of a knee brace. In the protocols in which ROM and WB are restricted, the brace is indicated for a 3‐ to 6‐week post‐operative period. In more accelerated protocols, the use of the brace is not routinely mentioned. The authors that mention it describe its use for the first post‐operative days in some cases or for the first weeks only to protect the knee during WB.

### After meniscus allograft transplantation

There are no high‐level studies available to guide the direction of rehabilitation in this setting. The literature focuses mainly on indications, techniques, outcomes, complications and RTS, with little being reported on the effect of rehabilitation management or differing strategies.

Nevertheless, experts' opinions and literature reviews make similar proposals. Patients who undergo meniscal allograft transplantation (MAT) are recommended to follow a dual restriction protocol [10], time‐based and criterion‐based. Biology and graft maturation may be of importance, so recovery may be slower than other meniscus procedures (meniscectomy or repairs) [14].

Rehabilitation is based on active and passive exercises depending on the time after surgery, with rehabilitation guidelines and goals being more delayed than accelerated [14]. WB is restricted for 6 weeks, and RTS is delayed to 9 months, if possible, at all, at a competitive level [20]. We acknowledge that there will be significant variability for each patient based on concomitant procedures, desired level of activity, and individual rates of recovery [8].

This consensus has some limitations.

This is not a systematic review and meta‐analysis of the entire literature on meniscus rehabilitation. The overall LOE of the literature covering meniscus rehabilitation is relatively low. The weight of the clinical expertise is higher when compared with scientific evidence on this topic. However, there are many strengths: This is an international worldwide consensus with a rigorous methodology based on five criteria (1—pluralism, 2—iterative process, 3—refereeing, 4—literature search and 5—transparency) which dramatically reduces the selection and confirmation biases which could appear [3].

There is (1) a large pluralism (more than 100 people involved) of the experts, selected according to precise rules. This is (2) an iterative process with totally independent groups working at different steps (steering and rating groups). These people, by their geographic distribution and medical specialties, do represent exactly the community‐target (3 Refereeing). Literature search (4) was performed according to precise rules and by an independent group. All the content of the search (literature summaries and reference list) is available on the website for complete transparency. Finally, this initiative has been done under the umbrella of established scientific societies with the assistance of a ‘neutral’ consensus projects advisor.

## CONCLUSION

Rehabilitation after meniscus surgery is a debated topic that may influence surgical outcomes if not optimally performed. Rehabilitation depends on the type of meniscus tear, the type of surgery, concomitant procedures, if any, and criterion‐based milestones.

This international EU‐US consensus provided an up‐to‐date overview of the best available evidence for clinicians (surgeons, physiotherapists, sports medicine doctors, etc.) treating meniscus‐injured patients. Take care of the meniscus!

More information about this consensus and the complete list of references can be found on the ESSKA website (https://esskaeducation.org/esska-consensus-projects) and on the ESSKA Academy Website (Open Access) https://esskaeducation.org/sites/default/files/2024-07/The%20formal%20EU-US%20Meniscus%20Rehabilitation%20Consensus.pdf.

## AUTHOR CONTRIBUTIONS

Nicolas Pujol, Airelle Giordano, Ben Ma and Robert Prill chaired the consensus and have been involved in all parts of the work. Stephanie Wong revised the first draft of this manuscript and was a member of the Steering Group. Phillipe Beaufils is the official ESSKA consensus projects advisor, who advised together with Juan Carlos Monllau Garcia adhering to the official consensus method. All other authors were members of the Steering Group and either developed questions and statements or did sufficient literature work. They are listed in alphabetical order. All authors were involved in the validation process of the statements. All authors read, revised and approved the final draft of this paper.

## CONFLICT OF INTEREST STATEMENT

Nicolas Pujol: Occasional consultant for education for Smith&Nephew, Stryker, ZimmerBiomet. Philippe Beaufils: ESSKA Consensus Projects advisor. Stephanie Wong: Editor‐in‐Chief of Current Reviews in Musculoskeletal Medicine. Robert Prill: Associate Editor KSSTA, Chair ESSKA Rehabilitation Committee. Airelle O. Giordano: Education Chair, AASPT. Robert F. LaPrade: Consultant: Smith and Nephew, Ossur; Royalties; Smith and Nephew, Ossur, Elsevier; Research support: AOSSM, AANA, Ossur, Smith and Nephew, Arthrex; Editorial Boards: AJSM, KSSTA, JEO, Journal of Knee Surgery; JISPT, OTSM; AOSSM: Nominating Committee; ISAKOS: Travelling Fellowship Committee, Program Committee. Aaron J. Krych: Consulting and Royalties Arthrex, Inc. Editorial Boards: AJSM, KSSTA. AANA Board. James J. Irrgang: Currently serves as President of the Board of Directors of Movement Media Sciences/Journal of Orthopaedic & Sports Physical Therapy. Alexandre Rambaud: Member of ESSKA Rehabilitation Committee; Deputy Editor: European Rehabilitation Journal, Journal de Traumatologie du Sport, Editor‐in‐Chief of Kinésithérapie, la Revue. Laura C. Schmitt: Editorial Board: MSSE; Research Support from Arthritis Foundation and NFL Players Association. Jill K. Monson: Member AASPT Research Committee, Consultant Smith & Nephew. Mark V. Paterno: Co‐Chair AASPT Research Committee, Research Support from AOSSM/AIrCast Research Foundation. Simone Perelli: Paid consultancy for Smith&Nephew. Aleksandra Królikowska: Member of ESSKA Rehabilitation Committee. Nicky van Melick: Member of ESSKA Rehabilitation Committee. Jitka Klugarová: Deputy director of the Czech CEBHC: JBI Centre of Excellence, Czech GRADE Network, member of methodological group within JBI and GRADE working group. The remaining authors declare no conflicts of interest.

## ETHICS STATEMENT

No need for systematic review and consensus statements not involving human participants.

## Supporting information

Supporting 1.

## References

[1] Alessio‐Mazzola M , Lovisolo S , Capello AG , Zanirato A , Chiarlone F , Formica M , et al. Management of ramp lesions of the knee: a systematic review of the literature. Musculoskelet Surg. 2020;104(2):125–133. 31595426 10.1007/s12306-019-00624-z

[2] Beaufils P , Becker R , Kopf S , Englund M , Verdonk R , Ollivier M , et al. Surgical management of degenerative meniscus lesions: the 2016 ESSKA meniscus consensus. Knee Surg Sports Traumatol Arthrosc. 2017;25(2):335–346. 28210788 10.1007/s00167-016-4407-4 PMC5331096

[3] Beaufils P , Dejour D , Filardo G , Monllau JC , Menetrey J , Seil R , et al. ESSKA consensus initiative: why, when and how? J Exp Orthop. 2023;10(1):101. 37801160 10.1186/s40634-023-00664-2 PMC10558408

[4] Beaufils P , Saffarini M , Karlsson J , Hirschmann MT , Prill R , Becker R , et al. High scientific value of consensus is based on appropriate and rigorous methodology: the ESSKA formal consensus methodology. Knee Surg Sports Traumatol Arthrosc. 2024;33:16–20. 39154255 10.1002/ksa.12390

[5] Cong T , Reddy RP , Hall AJ , Ernazarov A , Gladstone J . Current practices for rehabilitation after meniscus repair: a survey of members of the American Orthopaedic Society for Sports Medicine. Orthop J Sports Med. 2024;12(2):23259671231226134. 38639001 10.1177/23259671231226134 PMC11025434

[6] Culvenor AG , Girdwood MA , Juhl CB , Patterson BE , Haberfield MJ , Holm PM , et al. Rehabilitation after anterior cruciate ligament and meniscal injuries: a best‐evidence synthesis of systematic reviews for the OPTIKNEE consensus. Br J Sports Med. 2022;56(24):1445–1453. 35768181 10.1136/bjsports-2022-105495 PMC9726950

[7] Dias JM , Mazuquin BF , Mostagi FQRC , Lima TB , Silva MAC , Resende BN , et al. The effectiveness of postoperative physical therapy treatment in patients who have undergone arthroscopic partial meniscectomy: systematic review with meta‐analysis. J Orthop Sports Phys Ther. 2013;43(8):560–576. 23756350 10.2519/jospt.2013.4255

[8] Getgood A , LaPrade RF , Verdonk P , Gersoff W , Cole B , Spalding T , et al. International Meniscus Reconstruction Experts Forum (IMREF) 2015 consensus statement on the practice of meniscal allograft transplantation. Am J Sports Med. 2017;45(5):1195–1205. 27562342 10.1177/0363546516660064

[9] Glattke KE , Tummala SV , Chhabra A . Anterior cruciate ligament reconstruction recovery and rehabilitation: a systematic review. J Bone Jt Surg. 2022;104(8):739–754. 10.2106/JBJS.21.00688 34932514

[10] Grassi A , Bailey JR , Filardo G , Samuelsson K , Zaffagnini S , Amendola A . Return to sport activity after meniscal allograft transplantation: at what level and at what cost? A systematic review and meta‐analysis. Sports Health. 2019;11(2):123–133. 30638438 10.1177/1941738118819723 PMC6391552

[11] Jacquet C , Mouton C , Becker R , Koga H , Ollivier M , Verdonk P , et al. Does practice of meniscus surgery change over time? A report of the 2021 “THE MENISCUS” Webinar. J Exp Orthop. 2021;8(1):46. 34173909 10.1186/s40634-021-00365-8 PMC8236008

[12] Kim JS , Lee MK , Choi MY , Kong DH , Ha JK , Kim JG , et al. Rehabilitation after repair of medial meniscus posterior root tears: a systematic review of the literature. Clin Orthop Surg. 2023;15(5):740–751. 37811518 10.4055/cios21231 PMC10551690

[13] Kopf S , Beaufils P , Hirschmann MT , Rotigliano N , Ollivier M , Pereira H , et al. Management of traumatic meniscus tears: the 2019 ESSKA meniscus consensus. Knee Surg Sports Traumatol Arthrosc. 2020;28(4):1177–1194. 32052121 10.1007/s00167-020-05847-3 PMC7148286

[14] Lee DW , Lee JH , Kim DH , Kim JG . Delayed rehabilitation after lateral meniscal allograft transplantation can reduce graft extrusion compared with standard rehabilitation. Am J Sports Med. 2018;46(10):2432–2440. 30010388 10.1177/0363546518783732

[15] Lind M , Nielsen T , Faunø P , Lund B , Christiansen SE . Free rehabilitation is safe after isolated meniscus repair a prospective randomized trial comparing free with restricted rehabilitation regimens. Am J Sports Med. 2013;41(12):2753–2758. 24114748 10.1177/0363546513505079

[16] Mameri ES , Jackson GR , Gonzalez F , Kaplan DJ , Jawanda H , Batra A , et al. Meniscus radial tears: current concepts on management and repair techniques. Curr Rev Musculoskelet Med. 2023;16(5):182–191. 37157051 10.1007/s12178-023-09831-5 PMC10188782

[17] Milliron EM , Magnussen RA , A Cavendish P , P Quinn J , Dibartola AC , Flanigan DC . Repair of radial meniscus tears results in improved patient‐reported outcome scores: a systematic review. Arthrosc Sports Med Rehabil. 2021;3(3):e967–e980. 34195666 10.1016/j.asmr.2021.03.002 PMC8220631

[18] Nepple JJ , Block AM , Eisenberg MT , Palumbo NE , Wright RW . Meniscal repair outcomes at greater than 5 years: a systematic review and meta‐analysis. J Bone Jt Surg. 2022;104(14):1311–1320. 10.2106/JBJS.21.01303 35856932

[19] O'Donnell K , Freedman KB , Tjoumakaris FP . Rehabilitation protocols after isolated meniscal repair: a systematic review. Am J Sports Med. 2017;45(7):1687–1697. 28256906 10.1177/0363546516667578

[20] Rosso F , Bisicchia S , Bonasia DE , Amendola A . Meniscal allograft transplantation: a systematic review. Am J Sports Med. 2015;43(4):998–1007. 24928760 10.1177/0363546514536021

[21] Steineman BD , LaPrade RF , Haut Donahue TL . Loosening of transtibial pullout meniscal root repairs due to simulated rehabilitation is unrecoverable: a biomechanical study. Arthroscopy. 2019;35(4):1232–1239. 30871905 10.1016/j.arthro.2018.11.041

[22] Williams RA , Morrissey MC , Brewster CE . The effect of electrical stimulation on quadriceps strength and thigh circumference in menisectomy patients. J Orthop Sports Phys Ther. 1986;8(3):143–146. 18802237 10.2519/jospt.1986.8.3.143

[23] You M , Wang L , Huang R , Zhang K , Mao Y , Chen G , et al. Does accelerated rehabilitation provide better outcomes than restricted rehabilitation in post arthroscopic repair of meniscal injury? J Sport Rehabil. 2023;32(3):335–345. 36476967 10.1123/jsr.2022-0069

