# Supplementary material for: The formal EU‐US Meniscus Rehabilitation 2024 Consensus: An ESSKA‐AOSSM‐AASPT initiative. Part I—Rehabilitation management after meniscus surgery (meniscectomy, repair and reconstruction)
Source: Knee Surg Sports Traumatol Arthrosc. 2025 May 12;33(8):3002–13. doi: 10.1002/ksa.12674 (PMC12310086; doi:10.1002/ksa.12674)
Supplement: Supplementary file 1 — Supporting 1. [file KSA-33-3002-s001.docx]

Search String:

("return"[All Fields] OR "returned"[All Fields] OR "returning"[All Fields] OR "returns"[All Fields] OR "therap*"[All Fields] OR "precaut*"[All Fields] OR ("weight bearing"[MeSH Terms] OR "weight bearing"[All Fields] OR ("weight"[All Fields] AND "bearing"[All Fields]) OR "weight bearing"[All Fields]) OR "immobili*"[All Fields] OR ("activable"[All Fields] OR "activate"[All Fields] OR "activated"[All Fields] OR "activates"[All Fields] OR "activating"[All Fields] OR "activation"[All Fields] OR "activations"[All Fields] OR "activator"[All Fields] OR "activator s"[All Fields] OR "activators"[All Fields] OR "active"[All Fields] OR "actived"[All Fields] OR "actively"[All Fields] OR "actives"[All Fields] OR "activities"[All Fields] OR "activity s"[All Fields] OR "activitys"[All Fields] OR "motor activity"[MeSH Terms] OR ("motor"[All Fields] AND "activity"[All Fields]) OR "motor activity"[All Fields] OR "activity"[All Fields]) OR ("nonsurgical"[All Fields] OR "nonsurgically"[All Fields]) OR "brac*"[All Fields] OR ("therapeutics"[MeSH Terms] OR "therapeutics"[All Fields] OR "treatments"[All Fields] OR "therapy"[MeSH Subheading] OR "therapy"[All Fields] OR "treatment"[All Fields] OR "treatment s"[All Fields]) OR ("nonop"[All Fields] OR "nonoperative"[All Fields] OR "nonoperatively"[All Fields]) OR ("conservancies"[All Fields] OR "conservancy"[All Fields] OR "conservancy s"[All Fields] OR "conservation"[All Fields] OR "conservational"[All Fields] OR "conservations"[All Fields] OR "conservative"[All Fields] OR "conservatively"[All Fields] OR "conservatives"[All Fields] OR "conserve"[All Fields] OR "conserved"[All Fields] OR "conserves"[All Fields] OR "conserving"[All Fields]) OR "RTS"[All Fields] OR ("exercise"[MeSH Terms] OR "exercise"[All Fields] OR "exercises"[All Fields] OR "exercise therapy"[MeSH Terms] OR ("exercise"[All Fields] AND "therapy"[All Fields]) OR "exercise therapy"[All Fields] OR "exercising"[All Fields] OR "exercise s"[All Fields] OR "exercised"[All Fields] OR "exerciser"[All Fields] OR "exercisers"[All Fields]) OR "Non-operative"[All Fields] OR "physio*"[All Fields] OR ("sport s"[All Fields] OR "sports"[MeSH Terms] OR "sports"[All Fields] OR "sport"[All Fields] OR "sporting"[All Fields]) OR ("physical examination"[MeSH Terms] OR ("physical"[All Fields] AND "examination"[All Fields]) OR "physical examination"[All Fields] OR "physical"[All Fields] OR "physically"[All Fields] OR "physicals"[All Fields]) OR "rehab*"[All Fields]) AND "menisc*"[Title/Abstract]
